# Infections and cancer: debate about using vaccines as a cancer control tool

**DOI:** 10.1186/1750-9378-8-16

**Published:** 2013-05-04

**Authors:** Sam M Mbulaiteye, Franco M Buonaguro

**Affiliations:** 1 Division of Cancer Epidemiology and Genetics, National Cancer Institute, National Institutes of Health, Department of Health and Human Services, Bethesda, MD, USA; 2 Molecular Biology & Viral Oncology Unit, Dpt of Experimental Oncology, Istituto Nazionale Tumori “Fond Pascale”, Naples, Italy

## Abstract

In 2012, *Infectious Agents and Cancer* commissioned a thematic series collection of articles on Prevention of HPV related cancer. The articles have attracted wide interest and stimulated debate, including about the utility of vaccines in cancer control. The application of vaccines to cancer control fulfills a promise envisioned at the turn of the 20^th^ century when remarkable experiments showed that some cancers were caused by infections. This suggested the possibility of applying infection-control strategies to cancer control. Vaccines represent the most practical cost-effective technology to prevent wide human suffering and death from many acute infectious diseases, such as small pox or polio. Hitherto applied to control of acute fatal infections, vaccines, if developed, might provide a potent way to control cancer. The articles in the HPV thematic series show success in developing and applying a vaccine against human papilloma virus (HPV). A vaccine is also available against hepatitis B virus (HBV), which causes liver cancer. These vaccines augment the tools available to control the associated cancers. Scientific endeavor continues for six other cancer-associated infections, mostly viruses. Not surprisingly, debate about the safety of vaccines targeting cancer has been triggered in the scientific community. Questions about safety have been raised for those populations where other means to control these cancers may be available. Although it is difficult to quantify risk from vaccines in individuals where other cancer control services exist, it is likely to be low. Vaccines are much safer today than before. Technological advancement in vaccine development and manufacture and improved regulatory review and efficient distribution have minimized substantially the risk for harm from vaccines. Formal and informal debate about the pros and cons of applying vaccines as a cancer control tools is ongoing in scientific journals and on the web. *Infectious Agents and Cancer* encourages evidence-based discussion to clarify understanding of the role of vaccines in cancer control. In a similar vein, the journal will not consider anecdotal reports and rhetorical arguments because they are unlikely to inform policy, regulation, or the public.

## Editorial

In 2012, *Infectious Agents and Cancer* commissioned a thematic series collection of articles on Prevention of HPV related cancer. The articles have attracted wide interest and stimulated debate, including about the utility of vaccines in cancer control. The application of vaccines to cancer control fulfills a promise envisioned at the turn of the 20^th^ century when remarkable experiments showed that some cancers were caused by infections. Vaccines represent one of the most successful medical advances of all time. Vaccines prevent suffering and death from many acute infectious diseases for which a vaccine can be developed. The application of vaccines on a global scale has been dramatically expanded as modern knowledge, techniques, management and policy frameworks have led to more cost-effective production and delivery pipelines to populations who need them. For example, vaccination programs, mandated by law or applicable regulations, have diminished morbidity and mortality from many infectious diseases that previously were scourges and posed severe economic burdens (such as measles, polio, diphtheria, *Haemophilus influenzae* type b and pneumococcal infections). Global vaccination programs have eradicated smallpox and reduced poliomyelitis transmission to low levels such that eradication is feasible [1]. With this success, the possibility to control highly variable pathogens (i.e. HIV, influenza virus, malaria etc.) through vaccines has emerged. In the cancer field, the search for human cancer caused by infections, particularly viruses, is motivated, in part, by the possibility of preventing such cancers through vaccination. By the turn of the 21^st^ century, 8 human viruses, including Epstein-Barr virus, human papilloma viruses (HPV), hepatitis B and C virus (HBV and HCV), had been linked to cancer in humans. Vaccines have been developed and are being delivered to populations against two of these viruses - HBV and HPV. These vaccines are dramatically reducing the incidence of new infections and they are expected to dramatically reduce the risk of associated cancers, namely, liver and cervical.

**Figure 1:**
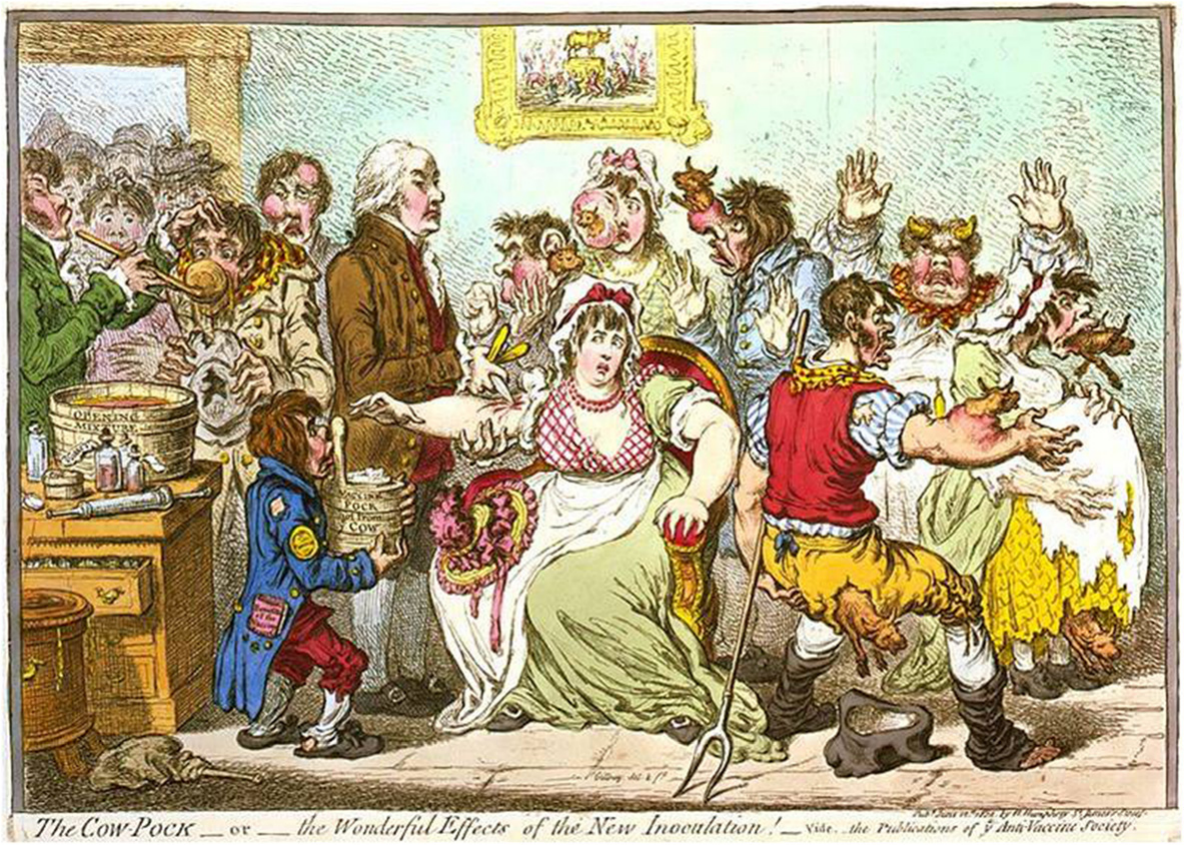
**The *Cow***-***Pock***—***or***—***the Wonderful Effects of the New Inoculation***! **caricature printed by James Gillray in 1802**, **humorously expressing the popular dread of vaccination.**

The science of vaccines and their application, however, has had a long history rooted in trial and error approaches, which may be relevant today. The “low dose” smallpox inoculation practices reported by Wang Zhangren's *Douzhen jinjing lu* (痘疹金鏡錄) in 1579 [2] and the introduction of “attenuated” cowpox vaccination to prevent small pox by Dr John Fewster in 1765 [3], standardized by Edward Jenner in 1798 [4], would probably not stand scientific scrutiny in today’s world. However, even at this time, fear of “inoculations” was already present, as noted in the cartoon from 1802 (Figure 1). A similar fear of vaccines exists today in some populations. Notwithstanding those fears, those pioneering efforts led directly to advances in knowledge and improvement in techniques to study immunity and to the development of vaccines, including the elimination of the arm-to-arm vaccinia transfer (associated with the high risk of syphilis transmission) and the worldwide implementation of the calf lymph vaccine developed by Galbiati in 1810 [5,6]. Pasteur’s discovery of an attenuated Rabies vaccine in 1885 [7] was a triumph against a frightening disease. The development of attenuated or inactivated vaccines against yellow fever, measles, rubella, and mumps, as well as the bacterial disease typhoid, Mycobacterium tuberculosis (BCG) Yersinia pestis (EV) followed in rapid succession. Attenuated vaccines induce transient growth of the virus in the host. This elicits durable immunity in the host and renders booster doses unnecessary. Attenuated vaccines, however, may cause harm when the attenuated viruses revert and become virulent again. In response, development of vaccines from inactivated – or killed- viruses eliminated the risk of reversion. Examples of inactivated vaccines include those used against influenza, cholera, bubonic plague, polio, hepatitis A, and rabies. Because transient growth does not occur with inactivated vaccines, booster doses are needed to elicit adequate immunity.

More recently, modern vaccines have relied on only components of the pathogen - the so-called subunits or conjugate vaccines. In some cases, these vaccines target inactivated toxins, such as the vaccines against tetanus and diphtheria. The subunits used are generally from a part of the pathogen exposed to the outside environment. Examples of subunit vaccines include the first generation of HBV vaccine, the current anti-HPV vaccines and *Haemophilus influenzae type B vaccine*. Since the’ 80s, innovation in vaccine technology has included production of subunit proteins synthetically by recombinant technology, which has replaced the crude method of extraction from the pathogen, and vastly improved the safety of modern vaccines.

Viewed in today’s terms, vaccines are distant from the blind “trial and error” inoculations of the 17^th^ or 18^th^ century. Today, vaccines are synonymous with prevention of morbidity and death from terrible diseases. Scourges of the past, like small pox or polio, have been subdued through vaccines. Vaccines represent hope, as well, to control more infections that still afflict millions, if not billions, worldwide. These include infections that cause human cancer, which are attributable to infections in 20-30% of cases. Vaccines have been developed against HBV and HPV, suggesting that cautious optimism may be warranted.

Nonetheless, as in the early days, the safety of vaccines remains a subject to intense debate. Caution is urged when deploying vaccines to identify and deal with life-threatening side effects that may emerge. Responsible discussion is also urged to tone down alarmist messages that may mislead the public to reject vaccines and increase their health risks.

Debate in the scientific literature is vital to identify and act on such concerns. It also provides neutral but authoritative reassurance to the general population. Specific questions related to individual vaccines arise from time to time and they need to be addressed in a sound and timely manner. Infectious Agents and Cancer is facilitating such a debate now. In their letter responding to an article by Serrano *et al*. on the anti-HPV nonavalent vaccine [8], Tomljenovic, *et al.*[9] question the safety of HPV vaccines and whether, if at all, they can be recommended for cervical cancer prevention. In her response, Dr de Sanjosè, a leading epidemiologist on HPV and guest Editor for the HPV Series, has provided robust responses to the questions raised by Tomljenovic, *et al*. [9]. The issues emerging from the current HPV debate, but relevant for vaccines against other oncogenic viruses, can be summarized as follows:

1) Given that cancer takes several decades to develop, can intermediate states in the pathway to cancer be used to evaluate the efficacy, and recommend the use, of anti-cancer vaccines?

2) Given that there may be dangers associated with vaccination, including the anti-HPV vaccines, do the dangers outweigh the benefits?

The scientific community addresses the first issue and regulatory Agencies address the second issue.

For anti-HPV vaccines, scientists have carefully considered virologic (i.e. virus isolation, virus titer) and cytological/histological end-points (i.e. pre-invasive and invasive lesions) and the natural history of disease. They have identified intermediate states, called pre-malignant end-points, which occur in the long interval between exposure to infection and cancer development. de Sanjosè and others explain this point adequately. A reduction of pre-invasive lesions represents a reliable intermediate end point for anti-HPV vaccines.

To weigh benefits versus risks requires unequivocal evidence of causation. HPV infection is necessary for HPV-related cancers to develop. This conclusion is based on evidence adduced by independent panels constituted by the International Agency for Research on Cancer, a part of the World Health Organization [10]. This conclusion is the basis for the scientific investment in studies designed to interrupt HPV transmission targeting the most important viral subtypes for cancer, so called high-risk genotypes, e.g., HVP 16 and 18. After many years of careful study, scientists have accurately estimated the efficacy of anti-HPV vaccines. Observational studies, using cancer registry data, may provide indirect evidence of efficacy against cancer by showing sustained reduction in cancer in ten-fifteen year time frame.

In the letter by Tomljenovic *et al.*[9], the authors question the logic of vaccinating girls to prevent long-term risks against cervical cancer. They assert that it is wrong to justify the vaccination against oncogenic virus to a pre-invasive cancer endpoint. They assert that vaccines unnecessarily expose young girls to harm. They question the entire process of vaccine development as being heavily tilted against unsuspecting vaccine recipients. However, questions of beneficence of vaccines have been exhaustively discussed before and will continue to be. P. Offit, in his article in the New England Journal of Medicine [11] and, in much greater detail, in his book *Vaccinated*: *One Man*’*s Quest to Defeat the World*’*s Deadliest Diseases* (HarperCollins, 2007) [12], outlines the quest to subdue disease through vaccination. As discussed above, the case of chronic infections introduces a further degree of complexity. It requires the use of validated intermediate states for cancer to evaluate vaccine efficacy. The intermediates states are very early stages of cancer initiation and progression. The intermediate states are already incorporated into cancer control strategies that rely in screening healthy women, identifying those with intermediate states and offering them early cancer care. It is therefore reasonable to assume demonstration that administering anti-HPV vaccines to young women reduces HPV infection and associated intermediate states indicates that the vaccine will ultimately have a positive impact on cancer morbidity. The intermediate endpoints can be to screen and identify individuals at high risk for cancer who can be offered specific treatments to prevent cancer as an alternative. Some countries, such as Italy and Australia, have adopted a policy for universal free vaccination at 11-years. Recent data from Australia for the period 2004 to 2011 demonstrated significant declines in the proportion of young women found to have genital warts and the no new cases of genital warts in vaccinated women [13]. These results show clearly that the human papillomavirus vaccine has a high efficacy outside of the trial setting and its addition to the already available cervical cancer control strategies, such as screening services will yield dividents. HPV vaccination of older women for cancer control is controversial and not widely recommended [14].

In summary vaccination technology coupled with vast improvements in distribution infrastructure had helped reduce morbidity and mortality from acute infections, and is poised to play a major role in cancer control. Improvements in recombinant technology and better choice of adjuvant formulations have made vaccines much safer [15,16]. Safety of vaccines will remain a matter of concern for many and debate will continue to inform scientists, public health managers, regulators, and users. Infectious Agents and Cancer welcomes and encourages reasonable debates with substantial data and authoritative reviews, which can be considered for publication.
